# Dietetics

**Published:** 1907-02-02

**Authors:** 


### DIETETICS.

In spite of our increasing knowledge of the metabolic processes of the human organism, we are still very much in the dark in regard to the influence of diet upon conditions of health and disease. Physicians are often led to dogmatise on questions of diet on a very slender basis of facts, and we find the most contradictory diets advocated, by different authorities, for one and the same morbid state. A vegetarian diet and also an exclusively meat diet, for example, have each found their advocates for the rheumatoid diathesis, and favourable results have been reported from each line of treatment. The truth seems to be that it is only within certain narrow limits that we are able to exercise any influence upon the intracellular chemistry of the organism by the nature of the food we present for absorption from the stomach and intestines.

Dietetic discussion now largely centres around the question of excess of food, and especially of the increased consumption of meat by the community. The consumption of meat in this country has risen from three pounds a head per annum in 1850 to fifty pounds a head per annum in 1902. It has been suggested that many evils which have increased, or are supposed to have increased, during this period, such as the greater frequency of cancer, the diminished birth-rate, and the spread of dental caries, may be attributed to this greater abundance of meat in the dietary of the community.

Professor Chittenden's work and views on the subject are already well known. He showed that soldiers and students leading an active life were able to keep in good bodily health, and to retain their weight, on a diet of very much smaller proportions, especially in proteid constituents, than was previously accepted generally, even by physiologists, as necessary. These results have been rather seriously impugned by Professor Benedict, of the Wesleyan University, Connecticut, who shows that the work done by the subjects of experiment, estimated in calories, exceeded the energy supplied by the food, and must therefore have entailed loss of body-weight. He further points out that the athletic students for the most part returned to their former diet after the experiments. They would hardly have done this if the experimental diet had been found to increase their chances of winning events for their University.

We are taught by physiologists that we should depend upon carbohydrates and fats for the fuel by the combustion of which we obtain the necessary energy for muscular exertion and other life processes, the proteid being requisite for the growth and repair of the tissues. Accepting this theory, it is not difficult to show that the usual consumption of proteid is far in excess of the needs of the organism for tissue repair. The question then arises, Is this excess of proteid over and above the actual requirements of the body not only unnecessary, but harmful?

Dr. Chalmers Watson has endeavoured to contribute to the solution of this problem by studying the effects of an exclusively meat diet on rats. These investigations showed that an exclusive meat diet, especially when commenced very early in life, retarded the growth of the rats, reduced their powers of reproduction and lactation, increased the mortality in the second generation, and also led to certain definite changes in the thyroid gland. Dr. B. P. Watson, by a similar series of experiments on rats, confirms the effect of a meat diet on rats in reducing their powers of reproduction and lactation.

In America, where indulgence in food appears to be greater than indulgence in drink, the physician doubtless sees many such conditions arising from a too liberal diet, and Professor Chittenden's word of warning may be very necessary. In England, too, a physician practising among well-to-do people becomes impressed with the evils resulting from the overladen table and the overladen stomach. But there is also the other side of the picture. Medical men who attend the less fortunate portions of the community are only too familiar with those conditions of anæmia, malnutrition, neurasthenia, and general debility, in which a good meal three times a day would be of infinitely more service to the patient than any bottle of physic.

The value of the Weir-Mitchell treatment in neurasthenia, and of the sanatorium feeding in tuberculosis, are a direct negative to any wholesale denunciation of a liberal diet, proteid or otherwise. The comparison recently instituted by Dr. A. S. Arkle, of Liverpool, between children of different grades in society, shows the effect of insufficient food on the growth and development of the body and mind. A boy of good circumstances at eleven years, is equal physically to a boy of fourteen in the lowest grade of schools. In the Japanese Navy, when beri-beri had become a scourge of the severest nature, a more liberal diet was immediately followed by a wonderful reduction in the incidence and mortality of the disease, and by a marked increase in the physique and general well-being of the men. On the whole, then, we are led to believe that a community will be more efficient and suffer less of the "ills to which flesh is heir," on a full stomach than on an empty one.

